# Arrhythmogenic Right Ventricular Cardiomyopathy

**DOI:** 10.7759/cureus.31446

**Published:** 2022-11-13

**Authors:** Tsering Dolkar, Nway Nway, Abubaker M Hamad, Hardik Jain, Alix Dufresne

**Affiliations:** 1 Internal Medicine, One Brooklyn Health Interfaith Medical Center, New York City, USA; 2 Medicine, Memorial Hospital of Converse County, Douglas, USA; 3 Internal Medicine, Saba University School of Medicine, Devens, USA; 4 Cardiology, One Brooklyn Health Interfaith Medical Center, New York City, USA

**Keywords:** myocardial fibro fatty tissue, echo cardiogram, cardiomyopathy, right ventricle, arrhythmogenic

## Abstract

Arrhythmogenic right ventricular cardiomyopathy (ARVC), formerly called arrhythmogenic right ventricular dysplasia/cardiomyopathy (ARVD/ARVC), is a myocardial structural abnormality disease with clinical presentation of cardiac arrhythmia. It is characterized by the replacement of the myocardium with fibrofatty tissue. We present a case of a young male who met two major criteria for definite diagnosis of ARVC: early transition inverted t waves in lead V1-V4 and MRI showed right ventricle (RV) dyskinesia with RV ejection fraction (EF) < 40%, both satisfying the two major criteria of EKG and MRI required for definitive diagnosis.

## Introduction

Arrhythmogenic right ventricular cardiomyopathy (ARVC), formerly called arrhythmogenic right ventricular dysplasia/cardiomyopathy (ARVD/ARVC), is a myocardial structural abnormality disease with clinical presentation of cardiac arrhythmia. It is characterized by replacing the myocardium with fibrofatty tissue [1]. The prevalence of ARVC in the general population is approximately one in 5,000 [2]. However, the diagnosis of ARVC frequently requires a high degree of clinical suspicion because many electrocardiographic abnormalities mimic the pattern seen in an average person, and the disease usually involves only a patchy area of the right ventricle.

## Case presentation

A 26-year-old African American male with a past medical history of allergic rhinitis and recurrent tonsillitis was referred from the medical clinic to the cardiology clinic for complaints of persistent left-sided chest pain of a few weeks duration. The patient presented to the cardiology outpatient clinic with complaints of chest pain for six weeks. He described the chest pain as left-sided, sharp, 6/10 in intensity, and unrelated to positional changes, food, or exertion. The patient reported a similar prior history of episodic chest pain but never before did it persist for weeks. He denied any history of recent exertive exercises before the onset of chest pain. He had no family history of sudden cardiac death or coronary artery disease.

A review of the system was positive for mild dizziness and negative for syncope, palpitations, seizures, or unexplained falls. The patient was afebrile, with blood pressure (BP) 131/81 mmHg, pulse 67/min, RR 18/min, and oxygen saturation 98% on room air. The cardiorespiratory examination was routine. The rest of the physical examination was not significant. The patient was evaluated with relevant tests and imaging. Laboratory workups were unremarkable with negative troponins, normal thyroid function test (TFT), normal serum calcium, and normal brain natriuretic peptide (BNP). Chest radiography (CXR) was unremarkable.

**Figure 1:**
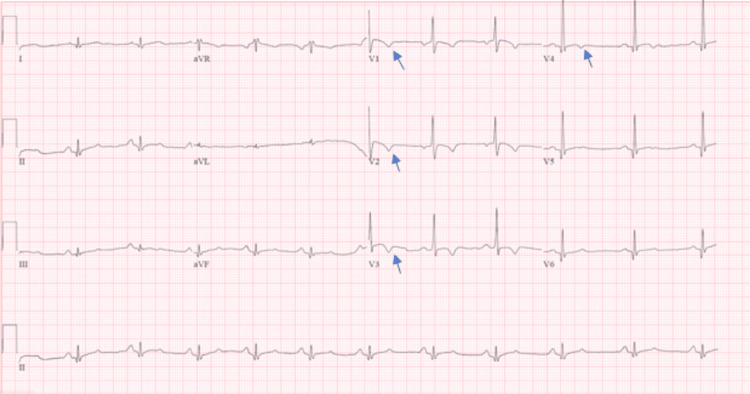
VR 67bmp, PR 160ms, QT 404ms, QTc 426ms, QRS 92ms. Normal sinus rhythm. Early transition T wave inverted V1-V4 (arrows).

EKG (Figure 1) showed normal sinus rhythm, rate of 67/min, interventricular conduction within normal limits, no limb criteria for hypertrophy, no ST elevations/depressions, QT interval of 404ms, QRS 92ms, QTc 426ms, no right bundle branch block (RBBB), no Wolf-Parkinson White (WPW) or Brugada pattern. Early transition T wave inverted in leads V1-V4.

The patient was scheduled for transthoracic echocardiography, which revealed normal left ventricular cavity size, ejection fraction 46-50%, apical inferior segment, basal segment, basal and mid inferolateral wall, and basal inferoseptal segment abnormality, normal left ventricular diastolic filling, dilated right ventricle, mildly dilated right atrium, and normal pulmonary artery systolic pressure. In addition, given regional wall motion abnormalities and dilated right ventricle, the patient was scheduled for a cardiac MRI and left heart catheterization.

**Figure 2:**
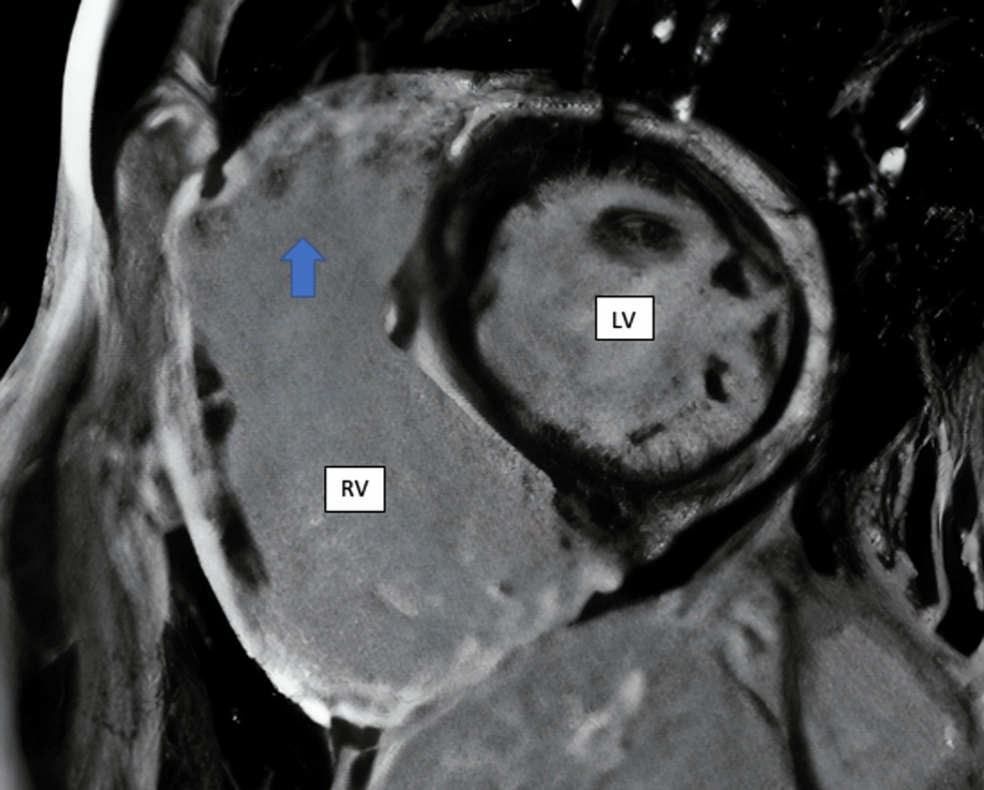
Cardiac MRI featuring dilatation of infundibulum of right ventricle (arrow). RV: right ventricle; LV: left ventricle

**Figure 3:**
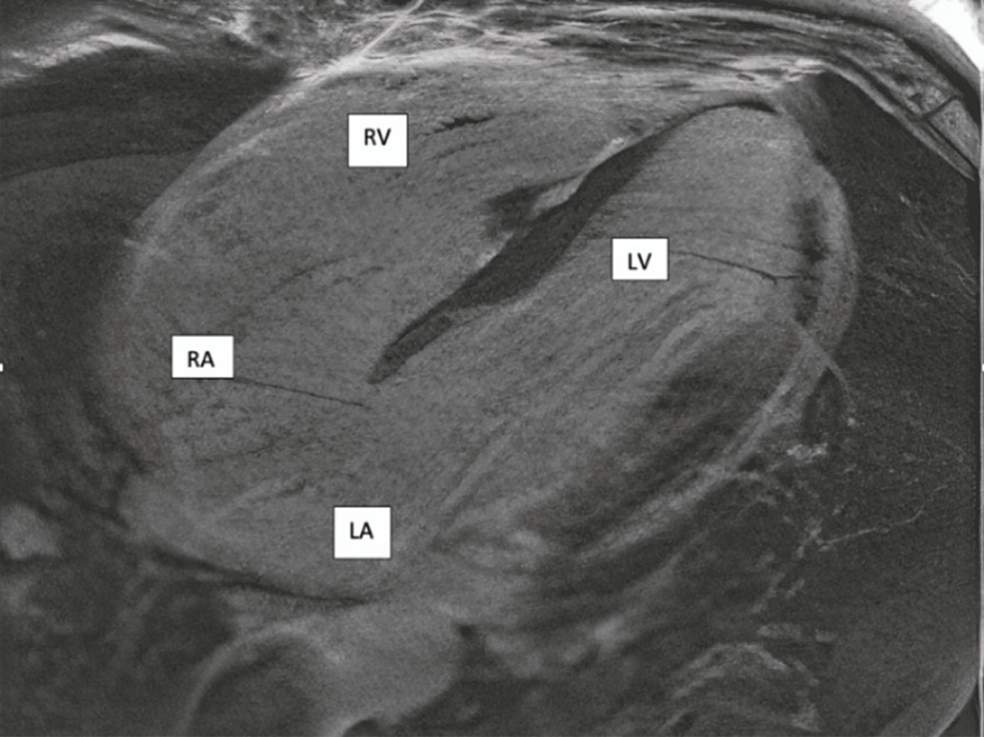
Cardiac MRI featuring marked dilatation of the right atrium (RA) and right ventricle (RV); showing left ventricle (LV) and left atrium (LA) for comparison.

**Figure 4:**
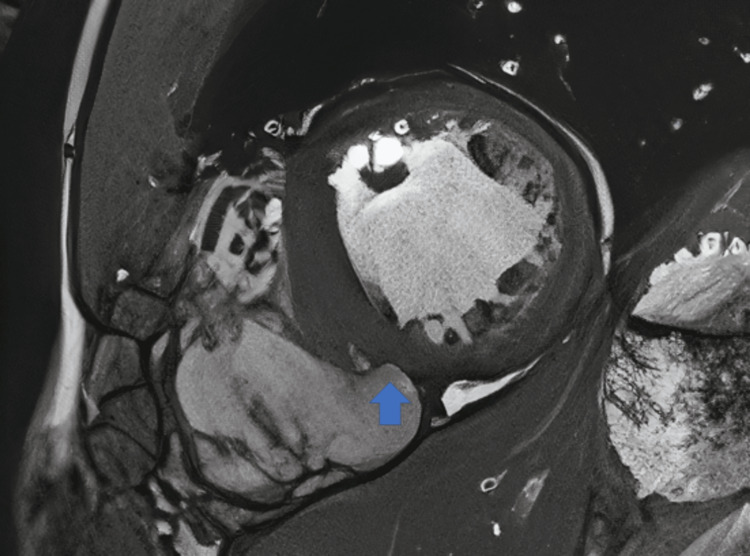
Cardiac MRI featuring focal aneurysm (arrow) measuring 1.9 cm at the infero/septal wall of the right ventricle.

The left heart catheterization revealed normal coronaries and possible mid-cavitary obstruction. Subsequently, a cardiac MRI was obtained and revealed dilatation of the infundibulum of the right ventricle measuring approximately 3.9 cm in the anteroposterior diameter (Figure 2), with marked dilatation of the right atrium and right ventricle (Figure 3), appearing lobulated with the presence of a focal aneurysm measuring 1.9 cm in the inferior/septal wall of the right ventricle. There was global hypokinesis, with this aneurysm appearing akinetic (Figure 4). The left atrium was not dilated; however, the left ventricle showed mild dilatation with mild global hypokinesis with no left ventricular hypertrophy. However, there were scattered regions of myocardial thinning in the septum, the lateral wall in the mid cavity, and involving the apex. In addition, there was delayed myocardial enhancement along the epicardial surface of the septum and involving the apical septal myocardium. The findings were suggestive of arrhythmogenic right ventricular dysplasia.

## Discussion

Diagnosis of ARVC

In 2010, Marcus et al. proposed revised criteria based on the original Task Force Criteria 1994. The diagnosis of ARVC as per Marcus et al. [3] is considered to be; (i) Definite: if the patient meets two major criteria OR one major criterion and two minor criteria OR four minor criteria from different categories; (ii) Borderline: if the patient meets one major criterion and one minor criterion OR three minor criteria from different categories; and (iii) Possible: if the patient meets one major criterion OR two minor criteria from different categories.

Diagnosis of ARVC/arrhythmogenic right ventricular dysplasia based on the revised Task Force Criteria include: (i) structural defects (global or regional), (ii) wall tissue characterization (myocardial wall replacement with fibrofatty tissue estimated in percentage upon endometrial biopsy); (iii) repolarization abnormalities (morphological changes of the T wave on the ECG); (iv) depolarization or conduction abnormalities (presence of epsilon waves on the ECG); (v) arrhythmias (ventricular tachycardia); (vi) family history (significant familial incidence of right ventricular arrhythmia).

Structural Defects: Global or Regional

Major Criteria: Severe dilation and decreased ejection fraction of the right ventricle.

• The 2D echocardiogram findings include regional akinesia, dyskinesia, or aneurysm in the right ventricle and one of the following findings during end-diastole: (i) parasternal window - long-axis views (PLAX) findings with right ventricle outflow tract findings of ≥ 32 mm, or (ii) parasternal window - short-axis views (PSAX) findings with right ventricle outflow tract findings ≥ 36 mm, or (iii) a change in the fractional area of ≤ 33%.

• MRI findings include regional akinesia, dyskinesia, or desynchronized contraction of the right ventricle and one of the following findings: (i) the ratio between right ventricular end-diastolic volume to body surface area of ≥ 110 ml/m^2^ in males or ≥ 100 ml/m^2^ in females; or (iii) an ejection fraction ≤ 40%.

• Right ventricle angiography findings include regional akinesia, dyskinesia, or aneurysm.

Minor Criteria: Mild global dilation and decreased ejection fraction of the right ventricle.

• 2D echocardiogram findings include regional akinesia, dyskinesia, or aneurysm in the right ventricle and one of the following findings during end-diastole: (i) PLAX findings with right ventricle outflow tract findings of ≥ 29 mm and < 32 mm; or (ii) PSAX findings with right ventricle outflow tract findings of ≥ 32 mm and < 36 mm; or (iii) a change in the fractional area of > 33% and ≤ 40%.

• MRI findings include regional akinesia, dyskinesia, or desynchronized contraction of the right ventricle and one of the following findings: (i) the ratio between right ventricular end-diastolic volume to body surface area of ≥ 100 ml/m^2^ and < 110 ml/m^2^ in males or ≥ 90 ml/m^2^ and < 100 ml/m^2^ in females; or (iii) an ejection fraction of > 40% and ≤ 45%.

Wall Tissue Characterization: Myocardial Wall Replacement With Fibrofatty Tissue Estimated In Percentage Upon Endometrial Biopsy

Major criteria include morphological myocyte deficit of < 60% and fibrous replacement of the right ventricular wall myocardium, and minor criteria include morphological myocyte deficit of > 60% but < 75% and fibrous replacement of the right ventricular wall myocardium.

Repolarization Abnormalities: Morphological Changes of the T Wave on the ECG

Major Criteria: T wave inversion in the right precordial leads - V1, V2, and V3 in patients more than 14 years old in the absence of a complete right bundle-branch block where the QRS segment is more than 120 ms.

Minor Criteria: T wave inversion in the right precordial leads - V1, V2 in patients more than 14 years old in the absence of right bundle-branch block or T wave inversion in leads V4, V5, or V6. Or T wave inversion in the right precordial leads - V1, V2, V3, and V4 in patients more than 14 years old in the presence of complete right bundle-branch block.

Depolarization or Conduction Abnormalities: Presence of Epsilon Waves on the ECG

Major Criteria: Reproducible low-amplitude epsilon waves are present between the QRS complex and the T wave onset in the right precordial leads, V1, V2, and V3.

Minor Criteria: Late potentials on the signal-averaged ECG with the absence of QRS duration of ≥ 110 ms on the standard ECG. Or Terminal activation duration of QRS ≥ 55ms measured from the nadir of the S wave to the end of the QRS, including R', in V1, V2, or V3, in the absence of a complete right bundle-branch block.

Arrhythmias: Ventricular Tachycardias 

Major criteria include sustained or non-sustained ventricular tachycardia with the left bundle-branch morphology with a superior axis, and minor criteria include sustained or non-sustained ventricular tachycardia of the right ventricular outflow configuration with the left bundle-branch morphology with the inferior axis, or more than 500 ventricular systolic beats on 24-hour Holter monitor readings.

Family History: Significant Familial Incidence of Right Ventricular Arrhythmia

Major Criteria: Based on the Task Force Criteria, the first-degree relative with ARVC/arrhythmogenic right ventricular dysplasia, or confirmation of anatomical pathology findings in the right ventricular surgery or autopsy in a first-degree relative. 

Minor Criteria: First-degree relative with suspected ARVC/arrhythmogenic right ventricular dysplasia where the Task Force Criteria have not been met or are impractical to be met; or sudden death of a first-degree relative younger than 35 years old, due to arrhythmogenic right ventricular cardiomyopathy/dysplasia; or second degree relative with suspected ARVC/arrhythmogenic right ventricular dysplasia based on the Task Force Criteria. 

Our patient meets the major criteria for definite diagnosis: one major criterion on EKG and one major criterion on MRI. The patient had early transition inverted T waves in lead V1-V4. MRI showed right ventricle dyskinesia with a right ventricle ejection fraction of less than 40%, satisfying the two major criteria required for a definitive diagnosis of ARVC. Most patients with ARVC experiencing arrhythmia have an abnormal EKG. A nonspecific or normal EKG does not exclude an ARVC diagnosis. Patients with nonspecific or normal EKG had alternative evidence of disease expression, which makes it more of a reason not to rely exclusively on EKG in ARVC but to opt-in for comprehensive evaluation [4]. Similar cases of ARVC in younger patients have been reported [5]. Though rare, ARVC is a common cause of sudden cardiac death in the younger population.

Management

Management involves pharmacologic therapy, radiofrequency ablation of an arrhythmogenic focus, and ICD. In patients with ventricular arrhythmias, amiodarone with or without beta-blocker are the most effective drugs combining the synergistic effect of class 3 antiarrhythmic with beta-blockade [6]. However, long-term amiodarone should be avoided, given the cumulative side effects. In patients with monomorphic ventricular tachycardia, catheter ablation is another therapeutic modality that can be employed.

Treatment reduces symptoms, limits disease progression, and prevents sudden cardiac death. Heart failure medications are used for patients with right or left-sided heart failures, such as beta-blockers, angiotensin-converting enzyme (ACE) inhibitors, or diuretics. Our patient is currently on metoprolol and is doing well.

Current guidelines include the following recommendations [7]: (i) Electrophysiological studies (EPS) should be considered in the diagnosis and/or evaluation of patients with suspected ARVC/arrhythmogenic right ventricular dysplasia (class IIa: weight of evidence/opinion is in favor of usefulness/efficacy.); (ii) Programmed ventricular stimulation may be considered for arrhythmic risk stratification of asymptomatic ARVC/arrhythmogenic right ventricular dysplasia patients (class IIb: usefulness/efficacy is less well established by evidence/opinion); (iii) Endocardial voltage mapping may be considered in the diagnostic and prognostic evaluation of ARVC/D patients (class IIb).

Radiofrequency ablation of an arrhythmogenic focus can be attempted in patients who are unresponsive or intolerant to antiarrhythmic drugs but is frequently unsuccessful and may require multiple attempts [8]. Patients at high risk for sudden cardiac death should receive an ICD. This includes patients who have been resuscitated from cardiac arrest, with a history of syncope or threatening arrhythmia that is not entirely suppressed by antiarrhythmic drug therapy.

## Conclusions

ARVC is increasingly recognized as a cause of malignant ventricular arrhythmia among apparently healthy young subjects and individuals engaged in vigorous exercise. Therefore, physicians should consider ARVC in young subjects with cardiac arrhythmia or unexplained cardiomyopathy. Periodic clinical assessment and non-invasive tests every two years are warranted, given the progressive nature of ARVC.
